# Acute behavioural disturbance: what's in a name?

**DOI:** 10.1192/bjo.2024.5

**Published:** 2024-02-01

**Authors:** Derek K. Tracy

**Affiliations:** Chief Medical Officer, West London NHS Trust, UK; Cognition, Schizophrenia and Imaging Laboratory, Department of Psychosis Studies, Institute of Psychiatry, Psychology and Neuroscience, King's College London, UK; Department of Psychiatry, Imperial College London, UK; and Department of Psychiatry, University College London, UK

**Keywords:** Acute behavioural disturbance, excited delirium, new psychoactive substances, transcultural psychiatry, patients

## Abstract

Acute behavioural disturbance (ABD) is a highly contentious topic, with debate about its validity as a construct. Particular concerns have been raised about how it places societal problems ‘in’ people – disproportionately from minority ethnic backgrounds – medicalising being a victim of violence. The author reflects on his experiences ‘with’ ABD.

This editorial complements the *BJPsych Open* paper by Polling et al,^1^ who explore the use of the term acute behavioural disturbance (ABD) across time in the records of a UK NHS Mental Health Trust, and argue that this is so diverse, inconsistent and lacking in evidence that ABD has become meaningless as a concept. Worse, these words and this label (it is not a diagnosis, despite what many think the ‘D’ stands for) can cause harm, particularly in minority populations.

That ‘mistaken D’ and its implications are interesting, important and tap into something deeper and profound in mental health, which we underestimate at our peril. Language, meaning and how we communicate matter more in psychiatry than most medical specialties, and it is a two-way street – speaking and listening. I reflect back on my own experience with ABD. I am a member of the Advisory Council on the Misuse of Drugs (ACMD), which advises the Home Office on drug harms. A few years ago, with a colleague from the ACMD, I had been discussing an apparent growth in behavioural disturbances linked with a plethora of new psychoactive substances (NPS; often misnamed – more problems with language and meaning – as ‘legal highs’). NPS are a somewhat niche interest of mine, although I have long argued that mental health professionals need to be better aware of a phenomenon that is widespread and considerably interfaced with psychiatric complications.^2,3^ Here, we seemed to have a concerning physiological problem linked with NPS. My colleague, an emergency department consultant, saw it in his workplace, and there were concerns about its appearance in psychiatric wards. Failure to treat – and the treatment is known – risked serious physical compromise, and even death. The name of the phenomenon is ABD, although even at that time this was disputed, and some earlier authors had spoken instead of ‘excited delirium’.

In 2021, we published a piece in *BJPsych Advances*, titled ‘Acute behavioural disturbance: a physical emergency psychiatrists need to understand’.^4^ It felt, we hoped, a useful update on a poorly understood issue. The list of conditions that can cause ABD is long, and beyond the remit of this editorial, but these range from illicit drug use to untreated mental illness, to cerebral pathology such as brain tumours. We highlighted physiological markers and a treatment algorithm to help reduce the likelihood of harm. Challenges were identified: the ‘D’ is not a ‘diagnosis’ and ABD is in neither the ICD nor the DSM. The symptoms listed are all possible, but none are pathognomonic or essential. Fundamentally, the validity of the construct is challenged. A modified version of this was subsequently included as part of the 14th edition of the Maudsley Prescribing Guidelines.^5^ So, the first part of communication was complete – speaking to others.

I did not really anticipate what would happen next. Some things in life are hard to predict, and others might have been foreseen with a little more thought and care. This was the latter. With hindsight, I had been a bit lost down my NPS rabbit hole, certainly wanting to shine light on an area I thought important and help to elucidate pathophysiological processes, but not stopping to take in a wider truth. In particular, I had not thought through the societal place of ABD, and how semantics and use of language actually carried far wider meaning in this particular case.

The context, as Polling et al point out, is that ‘meaning’ behind purported instances of ABD is often so wide as to actually lose any meaning at all. More pertinently, what lies beneath is a term commonly bandied about to cover any number or forms of agitation and/or physiological compromise, and something that – intentionally or not – is disproportionately used against individuals from minority ethnic backgrounds. Especially young Black men such as

George Floyd. There are two words that have meaning. As does ‘I can't breathe’, the last thing he said as he lay dying. ABD was mentioned in the trial of police officer Derek Chauvin in the USA, with debate as to this being a causative part of Floyd's death. However, George Floyd did not have an illness. He was murdered, and Chauvin was convicted. One can immediately see the issue of putting the ‘problem’ in the person, rather than contextualising it in the society in which we live. There have been reasoned arguments that ABD has been used inappropriately as a defence to hide behind brutality, and even worse, brutality amplified by institutional racism. One could argue that such racism, conscious or subconscious, infiltrates at any number of levels, from first-line emergency responders to treating clinicians, to scientific institutions carrying out research. A phrase that has been used in the literature for half a century has a very different complexion in 2024.

There are resonances with other discussions in mental health. Psychiatry has a complex history with labels and those to whom they are applied. One can think through historical examples such as ‘hysteria’, to more contemporary debate around borderline personality disorder (BPD). BPD makes a perhaps uncomfortably deep analogy with ABD, where many feel they have had this term (and another disputed ‘D’) weaponised against them, including concern for many about underpinning misogyny. Again, debate is not just about a label (whether it should be renamed complex post-traumatic stress disorder), but a more fundamental issue of how professionals understand and speak about and with people. This latter part is critical, as just changing a name, however disliked, risks being a meaningless platitude without any appropriate change in attitudes. Back to BPD, the argument from some is that this occurs when there is a shift from ‘what is wrong with you’ to ‘what happened to you’.

Arguments about ABD persist today. The inclusion of our work in the Maudsley Prescribing Guidelines was criticised in a national newspaper and condemned by some.^6^ The Royal College of Psychiatrists issued an initial critical statement on ABD in 2021, that was in turn rebutted by the Police Federation of England and Wales.^7^ This was followed by a partial retraction and more detailed guidance on the topic by the College (my declaration of interest is that I was one of the authors of this).^8^ The Royal College of Emergency Medicine subsequently updated its own guidance on the topic,^9^ but this differed considerably from the Royal College of Psychiatrists’, and also affirmed ABD as a condition needing appropriate clinical management. We all have our blinkers and failings. I think psychiatry is sometimes rightly accused of naval gazing and preoccupation with semantics. If I may leave my lane for a moment, a comment I heard by an emergency department consultant (not the co-author of the earlier mentioned piece) relating to ABD to ‘just treat the body’ was as unhelpfully abstract and isolated in its own way. My own opinion is that there has been inadequate communication by all parties in issuing their own guidance but failing to come together.

Where do we go from here? For me, I had not really listened and thought enough before I spoke, and I inadequately considered a wider world around me. I am sorry for that and for any upset I caused even inadvertently, and it is a lesson that has stayed with me. I am personally grateful to Lade Smith, the senior author of the linked paper, and now the President of the Royal College of Psychiatrists. She has given me thoughtful counsel and reflections on how words and language impact on those who have been affected by ABD, not least those from minority ethnic backgrounds, and in particular young Black men.

In my area of interest of NPS, I have concerns about some people developing harm from drug consumption, and I would like to educate clinicians about this. Ongoing toxicological work that I am engaged with, with colleagues in Taiwan, shows concerning and dangerous NPS-induced physiologically abnormal agitated presentations in the emergency department.^10^ But words matter, language matters. I am not sure that ABD as a term remains fit for purpose, and I am minded it is too heavily tainted by the weight of too many injustices to persevere. A recent UK-based multidisciplinary Delphi study^11^ has proposed some areas of consensus from participants from a range of professional backgrounds, including that ABD is not a separate entity to agitation and should be renamed. They have proposed key criteria to identify those at most risk of poor outcomes, specifically tactile hyperthermia (being hot to touch), constant activity and extreme agitation or aggression. It is not clear if these will be adopted by any organisations or bodies, and in the UK I think the Royal Colleges still need to come together with the police and others. People with lived experience need to be at the centre of this. Outstanding issues include the name, but as importantly, some attempt at consistency in agreeing what this encompasses, and by which criteria it is defined (even if not diagnosed). This would help clinical services, law enforcement, the coroner's office and those personally affected by the condition. Better data collection would lead to refinement of principles, understanding and treatment through some prospective research rather than the necessarily common, but inevitably heavily flawed, retrospective data analysis. None of us can stay siloed and not recognise wider perspectives. We talk of biopsychosocial, which of course is always really (or really should be) sociopsychobio, and we need to anchor within a societal perspective. I think we need to all adopt a position of humility, and in that two-way street of communication, start with the listening part.
